# Supplementary material for: Anxiety and depression among hypertensive patients during the COVID-19 pandemic: A cross-sectional study from Kathmandu Metropolitan, Nepal
Source: PLoS One. 2024 Jul 29;19(7):e0308048. doi: 10.1371/journal.pone.0308048 (PMC11285924; doi:10.1371/journal.pone.0308048)
Supplement: Attachment — Submitted filename: PONE-D-23-25764_reviewer.pdf [file pone.0308048.s001.pdf]

PLOS ONE

Anxiety and Depression among Hypertensive Patients during the COVID-19 Pandemic: A Cross-Sectional Study from Kathmandu Metropolitan

--Manuscript Draft--

**Manuscript Number:**	PONE-D-23-25764
**Article Type:**	Research Article
**Full Title:**	Anxiety and Depression among Hypertensive Patients during the COVID-19 Pandemic: A Cross-Sectional Study from Kathmandu Metropolitan
**Short Title:**	Anxiety and Depression among Hypertensive Patients during the COVID-19 Pandemic
**Corresponding Author:**	Durga Shrestha, Bachelor of Public Health<br>Pokhara University<br>Pokhara, Gandaki NEPAL
**Keywords:**	Anxiety; Depression; Hypertension; COVID-19
**Abstract:**	Introduction: The outbreak of a global pandemic like COVID-19 has caused significant distress around mental health. The burden of mental health issues like anxiety and depression is a serious public health issue that requires evidence-based intervention. The study aims to determine the prevalence of anxiety and depression and the factors associated with it among hypertensive patients.<br>Materials and Methods: Quantitative method and cross-sectional study design were used for this study. The study was conducted among 374 samples of selected wards of Kathmandu Metropolitan through a convenience sampling technique. A face-to-face interview was conducted using a structured interview schedule. A Chi-square test was used to identify the statistical significance between dependent and independent variables. Logistic regression was applied to identify the factors associated with anxiety and depression.<br>Results: The prevalence of anxiety and depression among hypertensive patients during the COVID-19 pandemic was 27.8% and 24.3% respectively. According to the result of bivariate logistic regression analysis, smoking/tobacco consumption, stayed in quarantine, positive COVID-19 test result, history of COVID-19 positive in family, History of death due to COVID-19 in family, visiting a hospital during the COVID-19 pandemic appeared to be powerful influencing factors of both anxiety and depression.<br>Conclusion: Our findings suggest that COVID-19 has a substantial effect on the mental health of hypertensive patients. This study highlights the need to develop early intervention and coping strategies among respondents to minimize the negative impact of COVID-19 on their mental health.
**Order of Authors:**	Dilasha K.C., Bachelor of Public Health
	Hari Prasad Kaphle, Master of Public Health, PhD
	Durga Shrestha, Bachelor of Public Health
	Nirmala Neupane, Master of Science in Nursing
**Additional Information:**	
**Question**	**Response**
**Financial Disclosure**<br><br>Enter a financial disclosure statement that describes the sources of funding for the work included in this submission. Review the submission guidelines for detailed requirements. View published research articles from *PLOS ONE* for specific examples.	The author(s) received no specific funding for this work.

Powered by Editorial Manager® and ProduXion Manager® from Aries Systems Corporation

This statement is required for submission and **will appear in the published article** if the submission is accepted. Please make sure it is accurate.<br><br>**Unfunded studies**<br>Enter: *The author(s) received no specific funding for this work.*<br><br>**Funded studies**<br>Enter a statement with the following details:<br>• Initials of the authors who received each award<br>• Grant numbers awarded to each author<br>• The full name of each funder<br>• URL of each funder website<br>• Did the sponsors or funders play any role in the study design, data collection and analysis, decision to publish, or preparation of the manuscript?<br>• **NO** - Include this sentence at the end of your statement: *The funders had no role in study design, data collection and analysis, decision to publish, or preparation of the manuscript.*<br>• **YES** - Specify the role(s) played.<br><br>* typeset	
**Competing Interests**<br><br>Use the instructions below to enter a competing interest statement for this submission. On behalf of all authors, disclose any competing interests that could be perceived to bias this work—acknowledging all financial support and any other relevant financial or non-financial competing interests.<br><br>This statement is required for submission and **will appear in the published article** if the submission is accepted. Please make sure it is accurate and that any funding sources listed in your Funding Information later in the submission form are also declared in your Financial Disclosure statement.<br><br>View published research articles from *PLOS ONE* for specific examples.	The authors have declared that no competing interests exist.

**NO authors have competing interests**<br><br>Enter: *The authors have declared that no competing interests exist.*<br><br>**Authors with competing interests**<br><br>Enter competing interest details beginning with this statement:<br><br>*I have read the journal's policy and the authors of this manuscript have the following competing interests: [insert competing interests here]*<br><br>* typeset	
**Ethics Statement**<br><br>Enter an ethics statement for this submission. This statement is required if the study involved:<br><br>• Human participants<br>• Human specimens or tissue<br>• Vertebrate animals or cephalopods<br>• Vertebrate embryos or tissues<br>• Field research<br><br>Write "N/A" if the submission does not require an ethics statement.<br><br>General guidance is provided below. Consult the submission guidelines for detailed instructions. **Make sure that all information entered here is included in the Methods section of the manuscript.**	Ethical approval was taken from the Institutional Review Committee (IRC), Pokhara University (Ref. No. 51/077/078). Permission to conduct the study was taken from ward office-26 of Kathmandu Metropolitan. Written or verbal informed consent was obtained from the participant since all the participants were not able to write. Confidentiality, anonymity, and privacy of information was maintained.

Powered by Editorial Manager® and ProduXion Manager® from Aries Systems Corporation

**Format for specific study types**<br><br>**Human Subject Research (involving human participants and/or tissue)**<br>• Give the name of the institutional review board or ethics committee that approved the study<br>• Include the approval number and/or a statement indicating approval of this research<br>• Indicate the form of consent obtained (written/oral) or the reason that consent was not obtained (e.g. the data were analyzed anonymously)<br><br>**Animal Research (involving vertebrate animals, embryos or tissues)**<br>• Provide the name of the Institutional Animal Care and Use Committee (IACUC) or other relevant ethics board that reviewed the study protocol, and indicate whether they approved this research or granted a formal waiver of ethical approval<br>• Include an approval number if one was obtained<br>• If the study involved *non-human primates*, add *additional details* about animal welfare and steps taken to ameliorate suffering<br>• If anesthesia, euthanasia, or any kind of animal sacrifice is part of the study, include briefly which substances and/or methods were applied<br><br>**Field Research**<br><br>Include the following details if this study involves the collection of plant, animal, or other materials from a natural setting:<br>• Field permit number<br>• Name of the institution or relevant body that granted permission	
**Data Availability**<br><br>Authors are required to make all data underlying the findings described fully available, without restriction, and from the time of publication. PLOS allows rare exceptions to address legal and ethical concerns. See the PLOS Data Policy and FAQ for detailed information.	Yes - all data are fully available without restriction

Powered by Editorial Manager® and ProduXion Manager® from Aries Systems Corporation

A Data Availability Statement describing where the data can be found is required at submission. Your answers to this question constitute the Data Availability Statement and **will be published in the article**, if accepted.<br><br>**Important:** Stating 'data available on request from the author' is not sufficient. If your data are only available upon request, select 'No' for the first question and explain your exceptional situation in the text box.<br><br>Do the authors confirm that all data underlying the findings described in their manuscript are fully available without restriction?	
**Describe where the data may be found in full sentences. If you are copying our sample text, replace any instances of XXX with the appropriate details.**<br><br>• If the data are **held or will be held in a public repository**, include URLs, accession numbers or DOIs. If this information will only be available after acceptance, indicate this by ticking the box below. For example: *All XXX files are available from the XXX database (accession number(s) XXX, XXX.*).<br>• If the data are all contained **within the manuscript and/or Supporting Information files**, enter the following: *All relevant data are within the manuscript and its Supporting Information files.*<br>• If neither of these applies but you are able to provide **details of access elsewhere**, with or without limitations, please do so. For example:<br><br>*Data cannot be shared publicly because of [XXX]. Data are available from the XXX Institutional Data Access / Ethics Committee (contact via XXX) for researchers who meet the criteria for access to confidential data.*<br><br>*The data underlying the results presented in the study are available from (include the name of the third party*	All relevant data are within the manuscript and its Supporting Information files.

and contact information or URL).<br>• This text is appropriate if the data are owned by a third party and authors do not have permission to share the data.<br><br>* typeset	
Additional data availability information:	

Manuscript Click here to access/download;Manuscript;Manuscript.docx

Full Title: Anxiety and Depression among Hypertensive Patients during the COVID-19 Pandemic: A Cross-Sectional Study from Kathmandu Metropolitan

Short Title: Anxiety and Depression among Hypertensive Patients during the COVID-19 Pandemic

Dilasha K.C.[1], Hari Prasad Kaphle[1], Durga Shrestha[1,*], Nirmala Neupane[1]

1 School of Health and Allied Sciences, Pokhara University, Pokhara-30, Kaski, Nepal.

*durgashrestha10.ds@gmail.com (DS)

// Abstract

Introduction: The outbreak of a global pandemic like COVID-19 has caused significant distress around mental health. The burden of mental health issues like anxiety and depression is a serious public health issue that requires evidence-based intervention. The study aims to determine the prevalence of anxiety and depression and the factors associated with it among hypertensive patients.

Materials and Methods: Quantitative method and cross-sectional study design were used for this study. The study was conducted among 374 samples of selected wards of Kathmandu Metropolitan through a convenience sampling technique. A face-to-face interview was conducted using a structured interview schedule. A Chi-square test was used to identify the statistical significance between dependent and independent variables. Logistic regression was applied to identify the factors associated with anxiety and depression.

Results: The prevalence of anxiety and depression among hypertensive patients during the COVID-19 pandemic was 27.8% and 24.3% respectively. According to the result of bivariate logistic regression analysis, smoking/tobacco consumption, stayed in quarantine, positive COVID-19 test result, history of COVID-19 positive in family, History of death due to COVID-19 in family, visiting a hospital during the COVID-19 pandemic appeared to be powerful influencing factors of both anxiety and depression.

Conclusion: Our findings suggest that COVID-19 has a substantial effect on the mental health of hypertensive patients. This study highlights the need to develop early intervention and coping

strategies among respondents to minimize the negative impact of COVID-19 on their mental health.

Introduction

The coronavirus disease 2019 (COVID-19) whose first case was identified in Wuhan, Hubei of China in December 2019 is an ongoing global pandemic that has caused several deaths and distressing situations worldwide [1]. The World Health Organization (WHO) declared it a Public Health Emergency of International Concern in January 2020 and a pandemic on March 11, 2020, also reports that as of 19th September 2020, the total confirmed cases of COVID-19 are 30,055,710 including 9,43,433 deaths [2].

The spread of this infectious disease has resulted in chaos even amongst the high-income countries which have adequate resources and well-administrated health systems. In a developing country like Nepal, where the government despite its updated policies hasn't been able to contain the widespread of the virus has faced a devastating loss in the economy. With the first case identified on 23rd January 2020 in Nepal [3], the confirmed cases have been rising ever since leading to more negative impacts and concerns on mental health.

A widespread occurrence of a new fatal disease can be overwhelming and create strong and stressful emotions that many may be unable to cope resulting in anxiety and depression. The WHO reports that the increase in mental health problems like depression and suicide is on the rise globally [4], in the light of this pandemic. It is also noted that in the case of Nepal, vulnerable groups, especially chronic disease patients are susceptible to infection and deaths due to COVID-19 [5]. The increasing trend of transmission has caused the fear of getting infected and losing a

loved one an enormous toll on people living with underlying conditions. The current scenario has caused panic and confusion among the general population as a result of a halt in normal lifestyle and also physical activity to some extent by temporary lockdowns imposed to contain the transmission of the disease [6].

The burden of mental issues like anxiety and depression amongst the risk group like hypertensive patients, during and aftermath of the COVID-19 pandemic is a serious public health issue. The crisis and its impact demand evidence-based intervention strategies and policies to combat the mental health effects of an already ongoing pandemic. This study is intended to identify the prevalence and factors associated with anxiety and depression amongst hypertensive patients due to the COVID-19 pandemic and contribute its effort to the formation of relevant policies.

Materials and methods

Study design

The study design was cross-sectional.

Study method

The study method was quantitative.

Study area

The study area was Kathmandu Metropolitan, Bagmati Province the capital city of Nepal. This site was selected based on the high prevalence of hypertension in Bagmati Province, which is reported in the STEPS Survey, 2019.

Study population

The study population was the adult population of age 40-59 years suffering from hypertension residing in Kathmandu Metropolitan, Bagmati Province.

Sample size

A sample size of 374 was determined based on the sampling formula $n = z^2 pq / E^2$ with a 95% confidence interval, 5% margin of error, and 0.42 % prevalence of hypertension in 40-60 years of population. The data was collected from 5th May, 2021 to 6th June, 2021.

Sampling technique

Non-probability convenience sampling technique was used for selecting the required sample for this study. Among the total of 32 wards, 2 wards were selected as per convenience. Probability proportional to size sampling was used to identify the required sample depending on the proportion of the population from the selected wards of the metropolitan.

Selection Criteria

Inclusion criteria

- The study includes all individuals aged 40-59 residing in selected wards and diagnosed with hypertension at least one year before the COVID-19 pandemic.

Exclusion criteria

- Individuals who were bedridden, not able to communicate, needed special care and suffered from chronic conditions like cancer, heart failure, and kidney failure.

Ethical considerations

Ethical approval was taken from the Institutional Review Committee (IRC), Pokhara University (Ref. No. 51/077/078). Permission to conduct the study was taken from ward office-26 of Kathmandu Metropolitan. Written or verbal informed consent was obtained from the participant since all the participants were not able to write. The participants were also made fully informed about the nature of the research in their local language. Confidentiality, anonymity, and privacy of information was maintained.

Research tools and their development

Socio-demographic variables, disease-related characteristics, lifestyle-related parameters, and COVID-19-related characteristics were obtained in the first section of the questionnaires.

In the second section of the questionnaire, Generalized Anxiety Disorder (GAD-7), a self-administered questionnaire was used, a tool to measure anxiety, developed by Robert L. Spitzer et al. The anxiety symptoms were calculated by assessing the GAD-7 tool where the score "0-4", "5-9", "10-14", and "≥15" indicates minimal, mild, moderate, and severe anxiety respectively. The minimal and mild were merged to indicate the absence of anxiety (≤10) whereas, moderate and severe were merged to show the presence of anxiety (≥10). Its total score ranges from 0 to 21 with a cut-off point of sensitivity of 89% and specificity of 82%.[7]

Lastly, Patient Health Questionnaire (PHQ-9), a self-administered questionnaire was used, a tool to measure depression, developed by Robert L. Spitzer et al. The depression symptoms were calculated by assessing the PHQ-9 tool where the score "0-4", "5-9", "10-14", and "≥15" indicates minimal, mild, moderate, and severe depression respectively. The Composite International Diagnostic Interview (CIDI) has validated Nepali PHQ-9 with a cut-off ≥10: sensitivity 0.94 and specificity 0.80. The minimal and mild were merged to indicate the absence of a depression (≤10) whereas, moderate and moderately severe were merged to show the presence of depression (≥10). It ranges from 0 to 27 and can be scored from 0 (not at all) to 3 (nearly every day). It has a sensitivity and specificity of 88%.[7]

Pretesting, validity, and reliability

The pretesting of the tools was done on 10% (37) of the sample size (374) to check the appropriateness of the questionnaire. The pretested data was entered, analyzed, and necessary modifications were done in the data collection tool. We made a few small modifications to the question wording based on pre-testing. The modifications were done in consultation with the supervisor. An extensive literature review and consultation were done with the supervisor to ensure validity. The interview schedule was back-translated (English-Nepali-English) for easy understanding.

Data collection technique

The data collection technique was a face-to-face interview using an interview schedule. After obtaining verbal or written consent, interviews with the qualified population were conducted. Before consent, the study's objective and purpose were clearly described. Confidentiality was also maintained. Data collection was conducted in the Nepali language.

Data Management and Analysis

Raw data was cleaned, coded, and entered by using EPI DATA version 3.1, and all the entered data was transferred to Statistical Package for Social Sciences (SPSS version 16) for further analysis, and data analysis was done by using a data analysis plan. Descriptive statistics were calculated for socio-demographic variables, disease-related characteristics, lifestyle-related parameters, and COVID-19-related characteristics as well as anxiety and depression. The chi-square test was used to identify the statistical significance between dependent and independent variables. Logistic regression was applied to identify the factors associated with anxiety and depression.

Results

Socio-demographic characteristics. Out of total 374 respondents, two-thirds of them were between the ages of 50-59 years old (75.1%) and male (75.7%). Most of the participants were from relatively advanced ethnic groups (91.2%). Almost half of the respondents had completed their secondary education (43.9%). The practice of owning a business (42.2%) was higher compared to other occupations and the maximum monthly income was noted to be up to Rs.20, 000 (33.4%). Most of the participants lived in a nuclear family (68.4%). (Table 1)

Table 1 Socio-demographic characteristics (n=374)

**Variables**	**Frequency (n)**	**Percentage (%)**
**Age (years)**		

40-49	93	24.9
50-59	281	75.1
Mean Age = 52.71 years, S.D. = 3.996, Minimum = 43 years, Maximum =59 years		
**Sex**		
Male	283	75.7
Female	91	24.3
**Ethnicity**		
Relatively disadvantaged groups	33	8.8
Relatively advantaged groups	341	91.2
**Education level**		
No formal education	15	4.0
Basic education	28	7.5
Secondary education	164	43.9
Undergraduate	34	9.1
Graduate/above	133	35.6
**Occupation**		
Service: Government/Private	142	38.0
Agriculture	14	3.7
Own Business	158	42.2
Daily wage labor	2	0.5
Foreign service	1	0.3
Housewife	57	15.2
**Monthly income**		

Up to 10,000	99	26.5
Up to 20,000	125	33.4
Up to 30,000	122	32.6
Up to 40,000	28	7.5
**Types of family**		
Nuclear	256	68.4
Joint/ Extended	118	31.6

Anxiety and Depression symptoms. Among the total participants, 27.8% of respondents were suffering from different forms of anxiety symptoms while 24.3% of participants were suffering from different forms of depression symptoms (Table 2)

Table 2 Anxiety and Depression symptoms (n=374)

**Variables**	**Frequency(n)**	**Percentage (%)**
**Anxiety**		
Minimal	38	10.2
Mild	232	62.0
Moderate	103	27.5
Severe	1	0.3
**Depression**		
Minimal	39	10.4
Mild	244	65.2
Moderate	91	24.3

Association of socio-demographic characteristics with anxiety and depression. None of the variables were found statistically significant with both anxiety and depression in Pearson's chi squire test. (Table 3)

Table 3 Association of sociodemographic characteristics with anxiety and depression

**Variables**	**Anxiety Symptoms**		**Chi-square**	**p-value**	**Depression Symptoms**		**Chi-square**	**p-value**
	**Yes (104; 27.8%)**	**No (270; 72.2%)**			**Yes (91; 24.3%)**	**No (283; 75.7%)**		
**Age**								
40-49 years	29(31.2)	64(68.8)	0.702	0.402	24(25.8)	69(74.2)	0.146	0.702
50-59 years	75(26.7)	206(73.3)			67(23.8)	214(76.2)		
**Sex**								
Male	84(29.7)	199(70.3)	2.036	0.154	72(25.4)	211(74.6)	0.779	0.378
Female	20(22.0)	71(78.0)			19(20.9)	72(79.1)		
**Ethnicity**								
Relatively disadvanta	9(27.3)	24(72.7)	0.005	0.943	7(21.2)	26(78.8)	0.191	0.662

ged groups								
Relatively advantaged groups	95(27.9)	246(72.1)			84(24.6)	257(75.4)		
**Education level**								
No formal education	100(27.9)	259(72.1)	0.010	0.920	5(33.3)	10(66.7)	0.688	0.407
Formal education	4(26.7)	11(73.3)			86(24.0)	243(76.0)		
**Types of family**								
Nuclear	68(26.6)	188(73.4)	0.626	0.429	58(22.7)	198(77.3)	1.237	0.266
Joint/Extended	36(30.5)	82(69.5)			33(28.0)	85(72.0)		

Association between disease's related characteristics and anxiety and depression. The family members with hypertension were significantly associated with anxiety ($p<0.005$) while none of the variables were found statistically significant with depression in Pearson's chi square test. (Table 4)

Table 4 Association of diseases related characteristics with anxiety and depression

Variables	Anxiety Symptoms	Chi-square	p-value	Depression Symptoms	Chi-square	p-value

	**Yes (104; 27.8%)**	**No (270; 72.2%)**			**Yes (91; 24.3%)**	**No (283; 75.7%)**		
**Duration of diagnosis**								
36-47 months	37(31.1)	82(68.9)	0.938	0.333	32(26.9)	87(73.1)	0.621	0.431
48-59 months	67(26.3)	188(73.7)			59(23.1)	196(76.9)		
**Another disease along with hypertension**								
Yes	12(28.6)	30(71.4)	0.014	0.907	12(28.6)	30(71.4)	0.462	0.497
No	92(27.8)	240(72.3)			79(23.8)	253(76.2)		
**Family members with hypertension**								
Yes	31(40.8)	45(59.2)	8.008	0.005*	23(30.3)	53(69.7)	1.823	0.177
No	73(24.5)	225(75.5)			68(22.8)	230(77.2)		
**(*statistically significant at p <0.05)**								

Association of lifestyle-related parameters with anxiety and depression. Smoking/tobacco consumption was highly significant ($p < 0.001$) with both anxiety and depression while moderate level physical activity and alcohol consumption were highly significant ($p < 0.001$) with anxiety only. (Table 5).

Table 5 Association of lifestyle-related parameters with anxiety and depression

Variables	Anxiety Symptoms		Chi-square	p-value	Depression Symptoms		Chi-square	p-value
	**Yes (104; 27.8%)**	**No (270; 72.2%)**			**Yes (91; 24.3%)**	**No (283; 75.7%)**		
**Smoking/Tobacco Consumption**								
Yes	49(53.3)	43(46.7)	39.377	<0.001*	36(39.1)	56(60.9)	14.514	<0.001*
No	55(19.5)	227(80.5)			55(19.5)	227(80.5)		
**Alcohol Consumption**								
Yes	74(36.1)	131(63.9)	15.531	<0.001*	54(26.3)	151(73.7)	0.995	0.318
No	30(17.8)	139(82.2)			37(21.9)	132(78.1)		
**Moderate Level Physical Activity**								
Yes	76(24.1)	240(75.9)	14.326	<0.001*	72(22.8)	244(77.2	2.648	0.104
No	28(48.3)	30(51.7)			19(32.8)	39(67.2)		
**(*statistically significant at p <0.05)**								

Association of COVID-19-related characteristics with anxiety and depression. All variables-stayed in quarantine, COVID-19 test result, stayed in isolation, history of COVID-19 positive in family,

history of death due to COVID-19 in family, visiting a hospital during COVID-19 pandemic were significantly associated with both anxiety and depression in Pearson's chi squire test ($p<0.05$). (Table 6)

Table 6 Association of COVID-19-related characteristics with anxiety and depression

**Variables**	**Anxiety Symptoms**		**Chi-square**	**p-value**	**Depression Symptoms**		**Chi-square**	**p-value**
	**Yes (104; 27.8%)**	**No (270; 72.2%)**			**Yes (91; 24.3%)**	**No (283; 75.7%)**		
**Stayed in quarantine**								
Yes	9(81.8)	2(18.2)	11.055	0.001*	9(81.8)	2(18.2)	6.435	0.011*
No	8(25.0)	24(75.0)			12(37.5)	20(62.5)		
**COVID-19 test result**								
Yes	66(41.3)	94(58.8)	8.633	0.003*	60(37.5)	100(62.5)	6.518	0.011*
No	22(23.2)	73(76.8)			21(22.1)	74(77.9)		
**Stayed in isolation**								
Yes	69(42.6)	93(57.4)	7.113	0.008*	63(38.9)	99(61.1)	8.894	0.003*

No	13(76.5)	4(23.5)			13(76.5)	4(23.5)		
**History of COVID-19 positive in family**								
Yes	74(33.3)	148(66.7)	8.309	0.004*	65(29.3)	157(70.7)	7.263	0.007*
No	30(19.7)	122(80.3)			26(17.1)	126(82.9)		
**History of death due to COVID-19 in family**								
Yes	18(54.5)	15(45.5)	12.889	<0.001*	15(45.5)	18(54.5)	8.771	0.003*
No	86(25.2)	255(74.8)			76(22.3)	265(77.7)		
**Visiting a hospital during the COVID-19 pandemic**								
Yes	33(42.3)	45(57.7)	10.322	0.001*	28(35.9)	50(64.1)	7.161	0.007*
No	71(24.0)	225(76.0)			63(21.3)	233(78.7)		
**(*statistically significant at p <0.05)**								

Bivariate logistic regression for factors having significant association with anxiety and depression. Smoking/tobacco consumption (OR: 4.703, CI.95%: 2.840- 7.789); (OR: 2.653, CI.95%: 1.590- 4.427), stayed in quarantine (OR: 13.500, C.I. 95%: 2.397- 76.037); (OR: 7.500, C.I. 95%: 1.382-

40.690), COVID-19 test result (OR: 2.330, C.I. 95%: 1.316- 4.125); (OR: 2.114, C.I. 95%: 1.183- 3.779), history of COVID-19 positive in family (OR: 2.033, C.I. 95%: 1.249- 3.310); (OR: 2.006, C.I. 95%: 1.203- 3.347), history of death due to COVID-19 in family (OR: 3.558, C.I. 95%: 1.719- 7.365); (OR: 2.906, C.I. 95%: 1.399- 6.036), visiting a hospital during the COVID-19 pandemic (OR: 2.324, C.I. 95%: 1.378- 3.919); (OR: 2.071, C.I. 95%: 1.207- 3.554) appeared as powerful influencing factors of both anxiety and depression respectively. (Table 7)

Table 7 Bivariate logistic regression for factors having significant association with anxiety and depression.

**Variables**	**Anxiety**		**Depression**	
	**UOR (95% CI)**	**p-value**	**UOR (95% CI)**	**p-value**
**Family members with hypertension**				
Yes	2.123(1.252-3.601)*	0.005*	1.468(0.839-2.568)	0.179
No	1		1	
**Smoking/Tobacco Consumption**				
Yes	4.703(2.840-7.789)*	<0.001*	2.653(1.590-4.427)*	<0.001*
No	1		1	
**Alcohol Consumption**				
Yes	2.617(1.609-7.4.258)*	<0.001*	1.276(0.790-2.060)	0.319
No	1		1	

**Moderate Level Physical Activity**				
Yes	2.947(1.657-5.243)	<0.001*	1.651(0.899-3.033)	0.106
No	1		1	
**Stayed in quarantine**				
Yes	13.500(2.397-76.037)*	0.003*	7.500(1.382-40.690)	0.020*
No	1		1	
**COVID-19 test result**				
Yes	2.330(1.316-4.125)*	0.004*	2.114(1.183- 3.779)	0.012*
No	1		1	
**Stayed in isolation**				
Yes	0.228(0.071-0.730)	0.013	0.196(0.061-0.627)	0.006
No	1		1	
**History of COVID-19 positive in family**				
Yes	2.033(1.249-3.310)*	0.004*	2.006(1.203 -3.347)*	0.008*
No	1		1	
**History of death due to COVID-19 in family**				
Yes	3.558(1.719-7.365)*	0.001*	2.906(1.399-6.036)*	0.004*
No	1		1	
**Visiting a hospital during the COVID-19 pandemic**				

Yes	2.324(1.378-3.919)*	0.002*	2.071(1.207-3.554)*	0.008*
No	1		1	
**(*statistically significant at p <0.05)**				

.

Discussion

Anxiety:

In this study, 27.8% of the respondents reported having anxiety during the COVID-19 pandemic. A previous study conducted in India also showed similar results i.e. 28%.[8] A study conducted among hypertensive patients in Ethiopia found a slightly higher prevalence rate (32%).[9] The findings from a study conducted in Hong Kong mentioned the deterioration in mental health during the COVID-19 pandemic to be 25.4%.[10] The consistent findings show that a public health emergency like the COVID-19 pandemic has substantially caused mental health issues among vulnerable groups like hypertensive patients. This might be due to the constant fear and uncertainty of communicable diseases like COVID-19 which has greater negative health impacts on vulnerable groups like hypertensive patients. Additionally, disruptions in healthcare services can lead to delays in managing hypertension, causing additional anxiety.

Factors associated with anxiety

This study showed that smokers/tobacco consumers are four times more likely to develop anxiety. A similar study conducted in Ethiopia also suggested a higher prevalence of anxiety among those who smokes/consumes tobacco.[9] Finding are quite usual as people with the habit of smoking/

tobacco consumption may have increased health concerns and respiratory vulnerability as smoking exacerbates cardiovascular risks and weakens the immune system. Furthermore, the anxiety is made worse by the inability to access healthcare facilities and the disruption of support for quitting smoking during the pandemic.

The findings in this study reported that staying in quarantine was 13.5 times more likely to be a factor of anxiety. A study conducted after the outbreak of coronavirus disease in 2019 suggested that quarantine during disease outbreaks can lead to frustration and boredom which could lead to issues like anxiety.[11] This may be due to the worry of catching the virus and managing their health as well as a lack of social connection and support during the pandemic.

This study revealed that both being tested positive for COVID-19 and family members tested positive for COVID-19 had a significant association with anxiety. Consistent with this finding, a study conducted in the Republic of Ireland reported that respondents whose family members had screened and confirmed the presence of COVID-19 had a higher level of anxiety.[12] It is noted that if a family member is ill due to the outbreak, it harms the respondents which can be exacerbated. Family members' health becomes a major concern, causing elevated anxiety over their state and likely outcomes. The personal health concerns of being diagnosed with COVID-19, increased vulnerability, and concerns about the virus's spread and contagiousness are exacerbated by the need to take precautions.

This study reported that respondents who had a family history of death due to COVID-19 were 2.9 times more likely to develop anxiety during the COVID-19 pandemic. Similarly, a study conducted among American adults[13] also reported that respondents who experienced the death of a loved one due to the pandemic are more likely to develop anxiety. This may be due to the isolation and

lack of support, complicated grief, health concerns, disruption of daily routines, and coping mechanisms that contribute to this vulnerability.

This study revealed that the respondents visiting a hospital during the COVID-19 pandemic were 2.071 times more likely to develop anxiety. The reason for this may be due to the fear of infection, COVID-19-related measures, limited social support, uncertainty and disruption of care, and heightened health concerns.

Depression

In this study, 24.3% of the participants reported having depression during the COVID-19 pandemic. A similar study conducted in India among all populations also suggested similar results, i.e. 25.1%.[8] Likewise, the findings of this study are consistent with that of a study in China which showed the prevalence of depression among hypertensive patients to be 26.8%.[14] The similarity in findings may be attributed due to similar sample sizes and study settings. However, a study conducted among hypertensive patients in Ethiopia found a lower prevalence rate, i.e. 5.73%.[9] The reason for this discrepancy could be due to differences in the study period.

Factors associated with depression

This study showed that those who smoke/consume tobacco are 2.6 times more likely to suffer from depression. Similarly, a study conducted in urban Nepal showed that smoking is 5 times more likely a factor for depression.[15] A study conducted in Turkey [16] suggested that respondents who tend to smoke have a moderate level of depression. Smoking/ tobacco consumption could be significantly associated with depression as people may use smoking/ tobacco consumption as a coping mechanism but over time it can actually cause more harm.

This study showed that those who stayed in quarantine were 7.5 times more likely to suffer from depression. Similar findings to ours were found in a study conducted among hypertensive patients.[17] Country-wide lockdown on the brink of a pandemic and enforcement of quarantine can lead to changes in normal daily routine, boredom, and loneliness which may result in depression among individuals.

The findings in this study have reported that both being tested positive for COVID-19 and family members with a history of COVID-19 positive result is associated with depression. Similar findings have been reported by a study conducted in Ireland [12] which shows that the confirmed case of COVID-19 in the family as well as on own self is associated with depression. This may be due to the greater vulnerability brought on by receiving a COVID-19 diagnosis, fear about the health of family members, and the emotional impacts of their experience.

This study reported a statistical association between the death of a loved one due to COVID-19 and depression. Similar to this finding, a study conducted among American adults[13] also reported that respondents who experienced the death of a loved one due to the pandemic are prone to depression. The isolation, complicated grief with not having the chance to do a proper last farewell to the loved one might have led to an increased chance of depression.

This study revealed that the respondents visiting a hospital during the COVID-19 pandemic were 2.34 times more likely to develop anxiety. This may be due to limited support, disruption of routine and healthcare, health concerns and uncertainty, and the overall emotional toll of the pandemic.

Limitations

This cross-sectional study, however, is not without limitations. The sample is representative of a population of hypertensive patients. Those who were excluded from this study were hospitalized

patients, who are at high risk of contracting COVID-19 and experience mental distress. Also, the data related to pre-existing diagnoses of anxiety and depression were not collected. The rapidly changing situation of COVID-19 is also likely to indicate a change in current results.

Conclusion

The study highlights that key factors like smoking/ tobacco consumption, staying in quarantine, being tested positive for COVID-19, having a history of COVID-19 positive in the family, death due to COVID-19 in the family, and hospital visit during the COVID-19 pandemic are associated with anxiety and depression among hypertensive patients during COVID-19 pandemic.

The findings obtained from this study conclude that hypertensive patients who are prone to mental health problems like anxiety and depression should be identified and screened and further provided with counseling and awareness to reduce the prevalence of anxiety and depression. It is further implied that those hypertensive patients who have stayed or are staying in isolation and quarantine should be continuously monitored and observed to minimize their association with anxiety and depression. Moreover, hypertensive patients who themselves have tested positive for COVID-19, have a family history of being positive for COVID-19, and suffered a loss of family members due to the COVID-19 pandemic should be reached with special care and attention including motivational sessions from health workers.

Acknowledgments

The authors wish to thank all the respondents for providing their consent and time to participate in the research and all the helping hands who helped either directly or indirectly during the conduction of the research.

References

1. Novel Coronavirus(2019-nCoV)Situation Report - 8 2020, January 28.: World Health Organization; 2020. Available from: https://www.who.int/docs/default-source/coronaviruse/situation-reports/20200128-sitrep-8-ncov-cleared.pdf?sfvrsn=8b671ce5_2.

2. WHO Coronavirus Disease (COVID-19) Dashboard 2020.: World Health Organization; 2020. Available from: https://covid19.who.int/table.

3. Health Sector Emergency Response Plan COVID-19 Pandemic. 2020, May.: Government of Nepal; 2020.

4. Policy Brief: COVID-19 and the Need for Actionon Mental Health. 2020, May: United Nations; 2020.

5. Situation report #222.: Government of Nepal; 2022.

6. Devkota HR, Sijali TR, Bogati R, Ahmad M, Shakya KL, Adhikary P. The impact of COVID-19 on mental health outcomes among hospital fever clinic attendants across Nepal: A cross-sectional study. PloS one. 2021;16(3):e0248684. Epub 2021/03/23. doi: 10.1371/journal.pone.0248684. PubMed PMID: 33750955; PubMed Central PMCID: PMCPMC7984647.

7. Sigdel A BA, Bhattarai N, Poon BC, Giri G, Marqusee H, et al. Depression, Anxiety and Depression-anxiety comorbidity amid COVID-19 Pandemic: An online survey conducted during lockdown in Nepal. medRxiv. 2020. PubMed Central PMCID: PMC20086926.

8. Verma S, Mishra A. Depression, anxiety, and stress and socio-demographic correlates among general Indian public during COVID-19. Int J Soc Psychiatry. 2020;66(8):756-62. Epub 2020/06/23. doi: 10.1177/0020764020934508. PubMed PMID: 32567466.

9. Edmealem A, Olis CS. Factors Associated with Anxiety and Depression among Diabetes, Hypertension, and Heart Failure Patients at Dessie Referral Hospital, Northeast Ethiopia. Behav Neurol. 2020;2020:3609873. Epub 2020/06/09. doi: 10.1155/2020/3609873. PubMed PMID: 32509038; PubMed Central PMCID: PMCPMC7246401.

10. Choi EPH, Hui BPH, Wan EYF. Depression and Anxiety in Hong Kong during COVID-19. International journal of environmental research and public health. 2020;17(10). Epub 2020/05/30. doi: 10.3390/ijerph17103740. PubMed PMID: 32466251; PubMed Central PMCID: PMCPMC7277420.

11. Brooks SK, Webster RK, Smith LE, Woodland L, Wessely S, Greenberg N, et al. The psychological impact of quarantine and how to reduce it: rapid review of the evidence. Lancet (London, England). 2020;395(10227):912-20. Epub 2020/03/01. doi: 10.1016/s0140-6736(20)30460-8. PubMed PMID: 32112714; PubMed Central PMCID: PMCPMC7158942.

12. Hyland P, Shevlin M, McBride O, Murphy J, Karatzias T, Bentall RP, et al. Anxiety and depression in the Republic of Ireland during the COVID-19 pandemic. Acta Psychiatr Scand. 2020;142(3):249-56. Epub 2020/07/28. doi: 10.1111/acps.13219. PubMed PMID: 32716520.

13. Gallagher MW, Zvolensky MJ, Long LJ, Rogers AH, Garey L. The Impact of Covid-19 Experiences and Associated Stress on Anxiety, Depression, and Functional Impairment in

American Adults. Cognit Ther Res. 2020;44(6):1043-51. Epub 2020/09/10. doi: 10.1007/s10608-020-10143-y. PubMed PMID: 32904454; PubMed Central PMCID: PMCPMC7456202.

14. Li Z, Li Y, Chen L, Chen P, Hu Y. Prevalence of Depression in Patients With Hypertension: A Systematic Review and Meta-Analysis. Medicine (Baltimore). 2015;94(31):e1317. Epub 2015/08/08. doi: 10.1097/md.0000000000001317. PubMed PMID: 26252317; PubMed Central PMCID: PMCPMC4616591.

15. Neupane D, Panthi B, McLachlan CS, Mishra SR, Kohrt BA, Kallestrup P. Prevalence of undiagnosed depression among persons with hypertension and associated risk factors: a cross-sectional study in urban Nepal. PloS one. 2015;10(2):e0117329. Epub 2015/02/12. doi: 10.1371/journal.pone.0117329. PubMed PMID: 25671522; PubMed Central PMCID: PMCPMC4324992.

16. Ustun G. Determining depression and related factors in a society affected by COVID-19 pandemic. Int J Soc Psychiatry. 2021;67(1):54-63. Epub 2020/07/02. doi: 10.1177/0020764020938807. PubMed PMID: 32605422; PubMed Central PMCID: PMCPMC7331110.

17. Sensoy B, Gunes A, Ari S. Anxiety and depression levels in Covid-19 disease and their relation to hypertension. Clinical and experimental hypertension (New York, NY : 1993). 2021;43(3):237-41. Epub 2020/11/13. doi: 10.1080/10641963.2020.1847132. PubMed PMID: 33176496.

Click here to access/download
Supporting Information
S1 file.sav
